# IP-10 Kinetics in the First Week of Therapy are Strongly Associated with Bacteriological Confirmation of Tuberculosis Diagnosis in HIV-Infected Patients

**DOI:** 10.1038/s41598-017-13785-3

**Published:** 2017-10-30

**Authors:** Alberto L. García-Basteiro, Edson Mambuque, Alice den Hertog, Belén Saavedra, Inocencia Cuamba, Laura Oliveras, Silvia Blanco, Helder Bulo, Joe Brew, Luis E. Cuevas, Frank Cobelens, Augusto Nhabomba, Richard Anthony

**Affiliations:** 1 Centro de Investigação em Saude de Manhiça (CISM). Rua 12, Cambeve CP 1929, Maputo, Mozambique; 2 ISGlobal, Barcelona Ctr. Int. Health Res. (CRESIB), Hospital Clínic - Universitat de Barcelona, Rossello, 132, 08036 Barcelona, Spain; 3 Amsterdam Institute for Global Health and Development (AIGHD), Academic Medical Center, Amsterdam, The Netherlands; 4 Institute for Life Sciences and Chemistry, HU University of Applied Sciences Utrecht, Utrecht, The Netherlands; 5 Tuberculosis reference laboratory, Center for Infectious Disease Research, Diagnostics and Perinatal Screening (IDS), National Institute for Public Health and the Environment (RIVM), P.O. Box 1, 3720 BA Bilthoven, The Netherlands; 6 Liverpool School of Tropical Medicine, Liverpool, UK

## Abstract

Simple effective tools to monitor the long treatment of tuberculosis (TB) are lacking. Easily measured host derived biomarkers have been identified but need to be validated in larger studies and different population groups. Here we investigate the early response in IP-10 levels (between day 0 and day 7 of TB therapy) to identify bacteriological status at diagnosis among 127 HIV-infected patients starting TB treatment. All participants were then classified as responding or not responding to treatment blindly using a previously described IP-10 kinetic algorithm. There were 77 bacteriologically confirmed cases and 41 Xpert MTB/RIF® and culture negative cases. Most participants had a measurable decline in IP-10 during the first 7 days of therapy. Bacteriologically confirmed cases were more likely to have high IP-10 levels at D0 and had a steeper decline than clinically diagnosed cases (mean decline difference 2231 pg/dl, 95% CI: 897–3566, p = 0.0013). Bacteriologically confirmed cases were more likely to have a measurable decline in IP-10 at day 7 than clinically diagnosed cases (48/77 (62.3%) vs 13/41 (31.7%), p < 0.001). This study confirms the association between a decrease in IP-10 levels during the first week of treatment and a bacteriological confirmation at diagnosis in a large cohort of HIV positive patients.

## Introduction

Tuberculosis (TB) remains a primary global health concern but the tools to diagnose and manage patients on long and complex treatment regimens are suboptimal. The World Health Organization (WHO) estimated that there were around 10.4 million new cases of TB in 2015^1^ but only two thirds of these were actually reported to the health authorities. Furthermore, only 57% and 15% of notified incident pulmonary and extra pulmonary TB cases respectively are ever bacteriologically confirmed^1^. Among this large group of clinically diagnosed patients, there is likely a proportion of cases who initiate treatment without having TB^2,3^, or receive first-line therapy but have drug resistant TB (DR-TB)^4^. Providing TB treatment to a patient without TB risks drug-related toxicity and delays the diagnosis of the true cause of symptoms. First-line treatment given to a patient with DR-TB could further select resistant mutants to the remaining active drugs and delays proper treatment. Thus, assays to confirm a TB diagnosis and to monitor TB treatment are needed for the appropriate management of patients^5^.

The methods routinely used to monitor TB treatment are sputum or culture conversion, resolution of radiological signs or clinical improvement^6^, which have considerable limitations. Sputum smear lacks sensitivity and is unable to document the viability of the bacilli. Culture is more sensitive but provides results after several weeks, while radiology and clinical improvement have low specificity. Under programmatic conditions sputum and culture conversion are performed at month 2 of treatment. Clinical improvement suggests the initial diagnosis was correct, although the poor specificity of symptoms and the coexistence of comorbidities make clinical monitoring relatively unreliable. Radiological improvement lacks specificity and is slow, requiring expert interpretation and, in many cases, might only become obvious after several months.

In recent years, several assays and strategies have been evaluated to monitor TB treatment. These include, sputum based strategies (early bactericidal activity (EBA) methods, new nucelid acid amplification tests (NAAT) or whole blood bactericidal activity), lung function, radiological markers (including X-Ray scores, ultrasound or PET/Computerized Tomography), transcriptomic profiling and host immune biomarkers (including interferon-gamma release assays [IGRAs])^6^.

The use of measurable host immune biomarkers has been reported to correlate with disease severity and could potentially be used at point of care^7^. These include acute phase proteins, cytokines, subsets of T-lymphocytes or matrix metalloproteinases activated from tissue destruction^6,8^. One of the most promising molecules whose kinetics has been associated with treatment response is IP-10 (CXCL-10), a pro-inflammatory chemokine involved in several pathological processes. IP-10 participates in the recruitment of activated T cells, macrophages and Natural Killer (NK) cells^9^. Increased serum IP-10 concentrations are found in patients with cancer, autoimmune diseases, active infections^10^ and active and latent TB^11–14^. Although most studies exploring the role of IP-10 to monitor TB treatment had small sample sizes (a few dozens in most cases), there seems to be a correlation between the time of sputum conversion and a decrease in IP-10 levels, both in fresh and dried plasma samples^15,16^.

Significant decreases in IP-10 are correlated with a good treatment response at month 2^17,18^ and correlate with sputum conversion^19^ and treatment outcome^20^. A pilot study in Nigeria and Nepal showed that the likelihood of culture positivity at diagnosis could be predicted through a change from high to low IP-10 plasma levels after seven days of treatment^21^.

We sought to validate this prediction in an HIV-infected population, which due to impaired immunity might show altered IP-10 responses. We investigated if changes in the kinetics of circulating IP-10 in the first week of therapy are associated with bacteriological confirmation of TB among adult HIV infected patients starting TB treatment. As a secondary objective we explored the relationship between serum IP-10 kinetics and treatment outcomes.

## Methods

### Study setting

The study was conducted in Manhiça district, Southern Mozambique, by the *Centro de Investigação em Saude de Manhiça* (CISM). Manhiça has approximately 178,000 inhabitants and 39,000 households^22^ and 39.9% of 18–47 year old adults have HIV^23^. TB notification rates in Manhiça have been rising since the late 1990s^1,24,25^ and TB mortality during treatment is very high, especially among HIV infected individuals^26^. Multidrug-resistant (MDR) TB prevalence in the district is 4% and 15% among new and retreatment cases, respectively^27^.

TB treatment for new patients is offered free of charge at all district health units, consisting of 6-month fixed dose combinations (isoniazid, rifampicin, ethambutol and pyrazinamide) which are replenished weekly. The initial diagnostic test used for HIV-infected patients with symptoms of TB is Xpert MTB/RIF®.

### Study design and procedures

Adults attending the HIV clinic at Manhiça Health Centre (MHC) with presumptive TB were consecutively enrolled in the study from August 2015 to March 2016. A presumptive TB case was defined as an adult ≥18 years old presenting with cough and/or weight loss and/or fever and/or night sweats of any duration. Potential participants were asked to sign an informed consent before enrolment. Inclusion criteria included a presumptive diagnosis of TB and documented HIV infection. Patients who had received TB treatment in the previous 6 months were excluded.

All participants with a TB diagnosis (regardless of bacteriological confirmation) were referred to the National TB Program (NTP) office at MHC to start treatment. Blood samples were collected before TB treatment initiation (D0), and on days 7 (D7) and 60 (D60). A window of +3 days (D7-D10) was permitted for the second visit and ±5 days for the final visit (D55-D65).

Patients were instructed to provide two sputum samples the day after first attendance to confirm the diagnosis of TB (as per national guidelines). Patients not providing sputum samples the next day were asked to provide a sputum sample on the spot before obtaining the blood sample and before initiating anti-TB treatment (ATT).

### Laboratory procedures

Five ml of blood were collected at D0 for CD4 counts and 2–5 ml on D7 and D60 using vacutainer tubes without anti-coagulant and transported to the local immunology laboratory for centrifugation at 1,500 rpm for 15 minutes. Sera were stored at −20 °C for maximum 4 weeks before IP-10 testing was performed.

Serum samples were tested using a commercial IP-10 ELISA (Becton Dickinson and Company, New Jersey, USA, - Human IP-10 ELISA Set. Cat. No. 550926) at 1:100 and 1:1000 fold dilution, following the manufacturer’s instructions. A standard curve was produced using freshly prepared serial dilutions of the kit’s reference standard from 500 pg/ml to 7.8 pg/ml.

All sputum samples were tested using Xpert MTB/RIF, liquid culture (BACTEC^TM^ MGIT^TM^ 960) and light smear microscopy (Ziehl Neelsen stain).

### Definitions and hypothesis

A TB case was defined as a person starting ATT, either microbiologically confirmed (positive light smear microscopy, Xpert MTB/RIF or culture) or clinically diagnosed as per WHO guidelines^28^. Treatment outcome was classified as cured, completed, failed, died, lost to follow up or not evaluated. Treatment success was defined as the sum of the outcomes of cured and completed.

**Figure 1:**
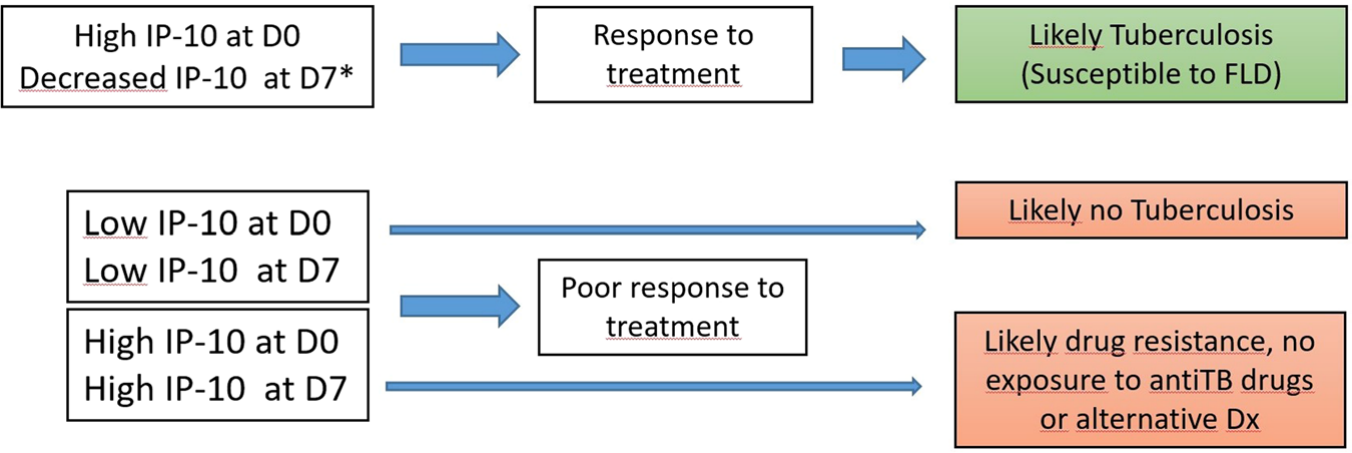
Treatment response classification algorithm used based on IP-10 Kinetics between D0 and D7.

We developed an algorithm to predict the expected TB treatment response prior to reviewing the observed diagnostic results and outcomes in the follow up. The outcomes predicted by the algorithm were blinded to all microbiological information and based only on the observed IP-10 kinetics on D0, D7 and D60 and on findings by den Hertog *et al*. (Fig. 1)^21^. Briefly, a D0 IP-10 > 1081 pg/ml followed by a decrease ≥300 pg/ml at D7 would predict a good ATT response. A D0 IP-10 > 1081 pg/ml without a significant IP-10 decrease (i.e. a change <300 pg/ml) on D7 would predict a poor TB treatment response (due for example to DR-TB, a disease other than TB, or lack of exposure to ATT, e.g. due to non-compliance or poor absorption). D0 IP-10 concentrations <1081 pg/ml were interpreted as no evidence of TB, regardless of the IP-10 value at D7.

All participants were then classified blindly using the IP-10 kinetic algorithm. We hypothesized that there would be a higher proportion of bacteriologically confirmed cases among those predicted to have “good IP-10 response” (Fig. 1).

### Data collection and analysis

Questionnaires were double entered into an electronic database. Data was then exported for analysis. ATT adherence was collected at D7 and D60 through self-reporting. Treatment outcomes were collected from the NTP registers at month 6. All deaths were confirmed through the CISM demographic surveillance system which covers nearly 100% of the district population. Data were analysed using STATA version 13 and R version 3.3.1 (Stata Corp, College Station TX, USA). Pearson’s Chi squared and Fishers’ exact tests were used to analyse the association between laboratory confirmation at diagnosis and IP-10 kinetics at D7 and D60 and Student’s T-test to compare means between different groups.

### Ethics statement

The study was approved by CISM’s Internal Scientific Committee, CISM Institutional Bioethics Committee for Health and the National Bioethics Committee of Mozambique (Reference 118/CNBS/14). All individuals provided written informed consent to participate. The study methods were carried out in accordance with the relevant guidelines and regulations established by the National Bioethics Committee at the Ministry of Health.

### Data availability statement

The datasets generated during the current study are kept at the data center of CISM. An anonymized version of the dataset can be made available upon request to CISM’s Internal Scientific Committee (Email: cci@manhica.net).

## Results

A total of 127 patients with a mean (SD) age of 37.1 (10.1) years of age were recruited. Seventy-four (58.3%) were male and 80 (63.0%) had bacteriologically confirmed TB. Of these, 4 had *rpo*B gene mutations by Xpert® MTB/RIF, although only 2 of these could be confirmed as MDR by Minimum Inhibitory Concentration (MIC) testing in liquid culture. Seven (9.2%) were mono-resistant to isoniazid by MIC. Over one third of the patients (34.3%) had CD4 counts <100 at recruitment. Twenty-eight (33%) participants died during the course of ATT and 72 (59.5%) had a successful treatment outcome (Table 1). Dying during treatment was more frequent in TB unconfirmed patients compared to those confirmed (16 (35.6%) vs 12 (15.8%), p value < 0.001). Blood samples on D7 and D60 were available for 118 and 101 patients, respectively.

**Table 1:** Baseline characteristics of 127 patients participating in the study.

Characteristics	n (%)
**TB case**	
Clinically diagnosed	47 (37.0)
Bacteriologically confirmed	80 (63.0)
smear	39(48.8)
MGIT culture (MTB)	74(92.5)
Xpert	79(99.0)
Resistance pattern (n = 76)*	
Resistant to rifampicin (Xpert) or MDR*	4 (5.2)
Resistant to isoniazid only	7 (9.2)
Resistant to isoniazid and pyrazinamide	1 (1.3)
Resistant to streptomycin only	4 (5.2)
Resistant to pyrazinamide only	2 (2.6)
**Sex**	
Male	74 (58.3)
Female	53 (41.7)
**CD4 counts/ml (n = 102)**	
<100	35 (34.3)
100-299	39 (38.2)
>300	28 (27.5)
**On Antiretroviral Treatment**	
Yes	75(61.5)
No	47(38.5)
**Age in years; mean (SD)**	37.1 (10.1)
**Treatment outcomes**	
Died	28(23.1)
Completed treatment	23(19.0)
Cured	49(40.5)
Failed	3(2.5)
Lost to follow up	13(10.7)
Not evaluated	5(4.1)

Acronyms: SD: standard deviation: Rif: rifampicin; MDR: multidrug resistant. *One rifampicin-resistant case (through Xpert) was not confirmed with MGIT based drug susceptibility testing. One other case with rifampicin resistance was contaminated in MGIT-based drug susceptibility testing.

All Rif susceptible patients started first-line intensive phase ATT (isoniazid, rifampicin, ethambutol and pyrazinamide) at D0 and the 4 Rif positive patients (detected through Xpert MTB/RIF®) started second-line treatment with kanamycin, ethionamide, levofloxacin, pyrazinamide, ethambutol and cycloserine.

### IP 10 kinetics

At D0, 87 (68.5%) of the 127 patients had IP-10 concentrations >781 pg/ml; 23 (48.9%) of 47 patients with clinically diagnosed TB and 16 (80.0%) of 80 bacteriologically confirmed cases had IP-10 > 781 pg/ml (p < 0.0001 using Pearson’s Chi-Squared test with Yate’s continuity correction). Among bacteriologically confirmed cases, patients on antiretroviral therapy (ART) were less likely to have IP-10 > 1081 pg/ml at D0 than patients not on ART (31/44 (70.5%) vs 28/31 (90.3%), p = 0.039). There was no statistically significant association between the D0 IP-10 classification and sputum smear status, CD4 count or sex.

**Figure 2:**
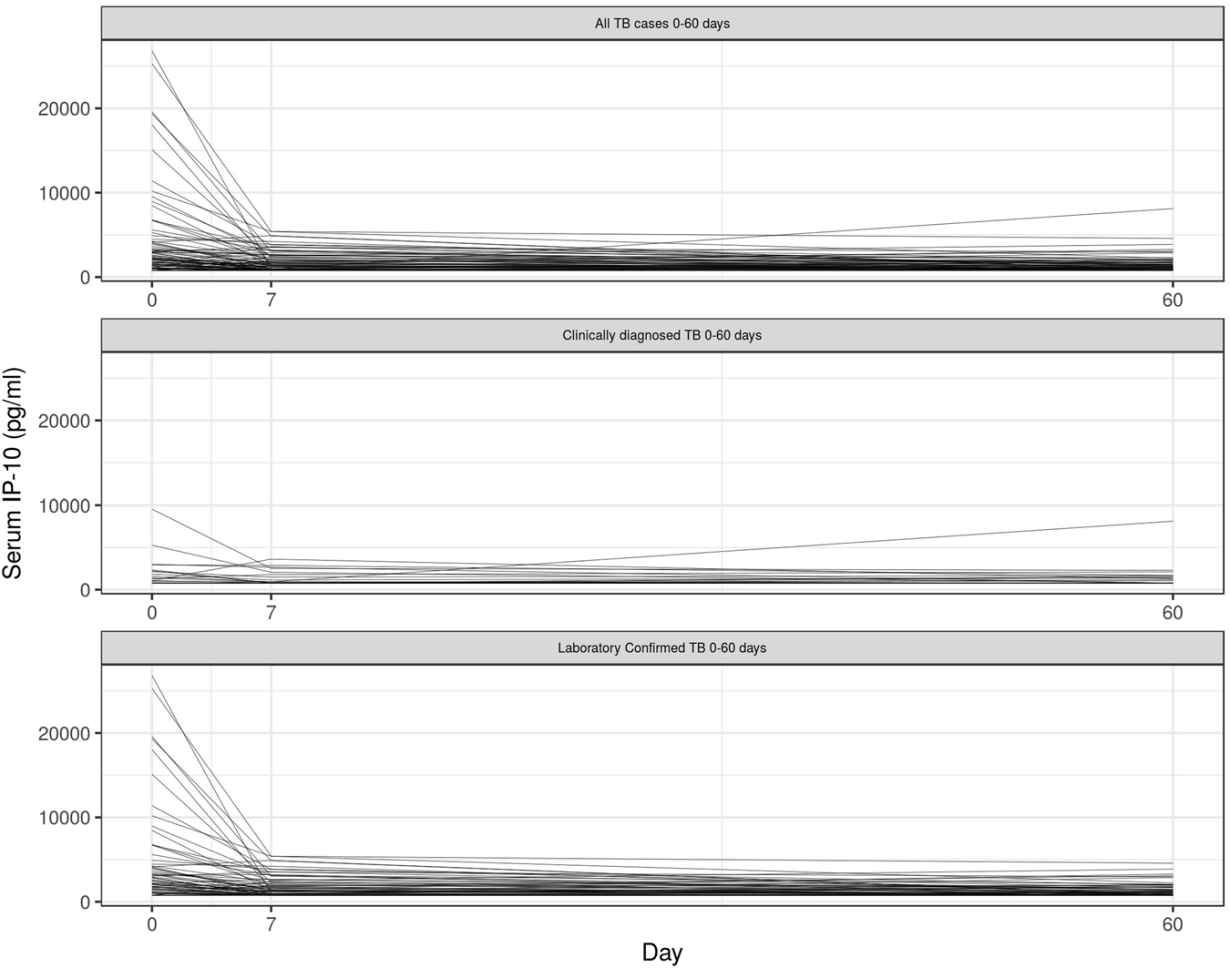
Kinetics of IP-10 at three time points (D0, D7 and D60). N = 126.

Figure 2 depicts the IP-10 kinetics on D0, D7 and D60. The majority of participants had a decline in IP-10 which occurred during the first 7 days of ATT. The mean decline of all participants between D0 and D7 was 1577 pg/dl. Bacteriologically confirmed cases were more likely to have high IP-10 levels at D0 and a steeper decline between D0 and D7 than clinically diagnosed cases (mean decline: 2282 vs 254 pg/dl for confirmed vs unconfirmed TB respectively; p = 0.002). No statistically significant declines between both groups were observed between D7 and D60 (486 vs 71 pg/dl for confirmed vs unconfirmed TB respectively, p value = 0.06).

### Prediction of TB laboratory confirmation based on IP-10 kinetics during the first week of treatment

With regard to the predictive algorithm, 61 patients had an IP-10 decline >300 pg/ml between D0 and D7. The proportion of bacteriologically confirmed cases was significantly higher among those who had an IP-10 decline (>300 pg/ml) than patients without significant IP-10 changes between D0 and D7 (48/61 (78.7%) vs 29/57 (50.9%), p = 0.002).

Among patients with no evidence of drug resistance, bacteriologically confirmed cases were 1.7 times more frequent among patients with IP-10 declines of >300 pg/ml in the first week than among patients with smaller IP-10 changes (38/51 (74.5%) vs 22/50 (44.0%) p = 0.002) (Table 2).

**Table 2:** Prediction of bacteriological status at diagnosis by IP-10 response between D0 and D7.

IP-10 based classification	Bacteriological status at diagnosis	All cases				Excluding cases with any drug resistance to HRZE^#^			
		Total cases*	n	(%)	p-value	Total cases	n	(%)	p-value
Response to treatment	TB bacteriologically confirmed	61	48	**78.7**	0.002	51	38	74.5	0.002
	TB clinically diagnosed		13	21.3			13	25.5	
Poor response to treatment	TB bacteriologically confirmed	57	29	50.9		50	22	44.0	
	TB clinically diagnosed		28	**49.1**			28	56.0	
**Total**		118				101			

*Some patients (n = 9) did not have D7 IP-10 data (including one Rif resistant case), thus were excluded in this table. ^#^This analysis includes cases with streptomycin resistance and excludes 4 cases bacteriologically confirmed without MGIT-based drug susceptibility testing. H:Isoniazid; R: Rifampicin; Z: Pyrazinamide; E: Ethambutol.

### Prediction of IP-10 kinetics performance based on bacteriological confirmation at D0

Bacteriologically confirmed cases were more likely to have a decline of IP-10 levels >300 pg/ml between D0 - D7 than clinically diagnosed cases (48/77 (62.3%) vs 13/41 (31.7%), p < 0.001). Similar results were obtained if only fully susceptible patients were included: 38/60 (63.3%) vs 13/41 (31.7%) for bacteriologically confirmed and clinically diagnosed cases respectively, p = 0.001) **(**Table 3 **)**.

**Table 3:** Prediction of treatment response based on IP-10 kinetics by laboratory confirmation status at D0.

Bacteriological status at diagnosis*		All cases			Excluding any case with drug resistance to HRZE^#^		
		n	%	p value	n	%	p value
**Bacteriologically confirmed TB (n = 77)**	Response to treatment (decline >300 pg/ml)	48	62.3		38	63.3	
	Poor response to treatment (decline <300 pg/ml)	16	20.8		13	21.7	
	Poor response to treatment (low IP-10 at D0)	13	16.3		9	15.0	
**Clinically diagnosed TB (n = 41)**	Response to treatment (decline >300 pg/ml)	13	31.7	<0.002^1^	13	31.7	0.002^1^
	Poor response to treatment (decline <300 pg/ml)	24	58.5	<0.001^2^	24	58.5	<0.001^2^
	Poor response to treatment (low IP-10 at D0)	4	9.8	0.012^3^	4	9.8	0.033^3^
**Total**		118			101		

*Some patients did not have D7 IP-10 data (=9), thus were excluded in this table. ^#^This analysis includes cases with streptomycin resistance and excludes 4 cases bacteriologically confirmed without MGIT-based drug susceptibility testing. H: isoniazid; R: rifampicin; Z: pyrazinamide; E: ethambutol. P-values correspond to the statistical significance between bacteriological status at diagnosis and IP-10 response (in a binary form): ^1^Response to treatment and Poor response to treatment (both “poor response” categories), ^2^Response to treatment and Poor response to treatment (decline <300 pg/ml); ^3^Response to treatment and Poor response to treatment (low IP-10 at D0).

Cases with IP-10 decreases >300 pg/ml (D0 - D7) were less likely to have death as the reported treatment outcome than patients with IP-10 differences <300 pg/ml (10/48 (20.8) vs 12/45 (26.7%), p value = 0.51). The same trend was observed if the analysis was restricted to bacteriologically confirmed cases (5/38 (13.2) vs 4/21 (19.0%), p-value = 0.34). However in both cases, this association was not statistically significant (Table 4).

**Table 4:** Association of treatment response based on IP-10 kinetics between day 0 and day 7 and treatment outcomes.

		Tuberculosis treatment response based on IP-10 Kinetics between D0 and D7											p-value
		All cases					p-value	TB Confirmed cases					
		Response to treatment		Poor Response to treatment		Total		Response to treatment		Poor Response to treatment		Total	
		n	%	n	%	n		n	%	n	%		
Treatment Outcome	Treatment success	38	79.17	33	73.33	71	0.51	33	86.84	17	81.0	50	0.34
	Died	10	20.83	12	26.67	22		5	13.16	4	19.0	9	
	Total	48		45		93		38	100	21	100	59	

## Discussion

This study builds on previous research assessing the role of IP-10 in TB treatment monitoring and confirms that IP-10 is also a promising serum biomarker to assess treatment response during the first week of treatment in patients with HIV. We report that serum IP-10 levels are generally increased before TB treatment and that increased levels are associated with bacteriologically confirmed TB. In our cohort of HIV-infected patients started on anti-tuberculosis treatment, IP-10 levels decreased during the early phases of treatment, as previously reported in HIV uninfected populations^15,17,18,21^. This study confirms that an IP-10 reduction >300 pg/ml in the first seven days of therapy is strongly associated with confirmed versus unconfirmed TB and that the absence of these changes is predictive of unconfirmed TB. Although IP-10 concentrations may reflect the bacteriological load in a patient, it is possible that a high proportion of clinically diagnosed patients did not have TB and were inappropriately treated, particularly as we used highly sensitive confirmation methods (both liquid culture and Xpert® MTB/RIF)^29,30^.

High IP-10 levels >1081 pg/ml on D0 as a single TB screening tool would have excluded 20% of confirmed TB cases, this performance is below what has been considered acceptable by a WHO panel^5^. This small but significant proportion of confirmed TB cases without dramatically raised IP-10 levels has been observed in other studies^15,21^. Investigating to what extent these patients differ from those with raised IP-10 levels needs to be elucidated. Among patients with a high IP-10 level at diagnosis, a limited or no decrease in IP-10 concentrations by D7 identifies patients who might be more likely to benefit from further diagnostic and clinical investigations. This approach may be especially valuable in settings with large proportions of patients with undiagnosed MDR-TB who would also be expected have a reduced/delayed IP-10 response to treatment.

Our blinded prediction of bacteriological status based only on IP-10 kinetics between D0 and D7 was correct in almost two thirds of the cases, and this association was statistically significant. Our hypothesis was quite simplistic since if IP-10 showed no response, we predicted it was from an unconfirmed patient or a drug-resistant case. However, even though we used a sensitive gold standard diagnostic we can be reasonably certain that a proportion of the clinically diagnosed patients in fact did not have TB^2,3^. Thus, we believe we present a minimum estimate of the performance of our predictive model and we would have been more accurate if there was a way of ascertaining the TB status among those who were clinically diagnosed. However, applying this algorithm to a population of TB presumptive cases who ultimately are diagnosed with another disease could further test our hypothesis. In addition, our hypothesis was that MDR patients would not show a decrease on IP-10 levels during the first week of treatment, but the number of MDR strains or Rif resistant strains in our sample was too low to test this assumption, and fact all of them received appropriate treatment. In addition, we do not know how the different levels of resistance or which specific drugs to which *M. tuberculosis* is resistant are necessary to affect the kinetics of IP-10. This was the reason why we conducted a subgroup analysis among those fully susceptible to first line drugs. However these results merely emphasize the need to assess IP-10 in settings with high prevalence of drug resistant TB, such as Eastern Europe.

Although those who showed a good response to treatment based in IP-10 kinetics between Day 0 and Day 7 died less frequently during treatment than those with poor response to treatment, there was no evidence that IP-10 kinetics in the first week of treatment predicts death during treatment (p-value > 0.05). Probably, many factors contribute to death during TB treatment, beyond an initial active therapy. Unfortunately, we do not know the actual cause of death among study patients under treatment, which would have been very interesting for this specific analysis.

In addition to the limited number of patients with drug resistance, our study had several other limitations. First, although the use of a commercially available ELISA kit (Becton Dickinson and Company, New Jersey, USA, - Human IP-10 ELISA Set. Cat. No. 550926) allowed us to perform this relatively large study onsite, it is different assay than that which was used in the earlier study^21^ we based our diagnostic algorithm on. This could mean that the cut offs used might not be exactly the same when using other commercially available assays^31^. The fact that similar conclusions can be reached using different assay formats is reassuring but may limit the generalizability of using those precise thresholds. In fact, it could be the case that the results we present are poorer than those that could be obtained with optimized cut off values for the assay used. Future head to head comparisons of different assays with same samples are needed to further validate or optimize the proposed cut offs.

Secondly, the ELISA kit and the serum dilutions measured resulted in a IP-10 detection limit of IP-10 limit of 780 pg/ml, thus some of the patients with an initial IP-10 level of <1081 pg/ml (allowing a decrease of 300 pg/ml to be quantified) might have been classified as “non-responders” due to the initially low IP-10 levels, despite having higher IP-10 levels than the average serum values in adult populations^32,33^. Thirdly, in this study we were not able to closely follow up patients beyond their month 2 visit. More detailed follow up could have identified alternative diagnoses or comorbidities explaining the initial TB-like symptoms, and contributed to a better characterization of the clinically diagnosed patients showing no response with regards to IP-10 kinetics. Lastly, our definition of good response was a decline of 300 pg/ml with an initial IP-10 level over 1081 pg/ml, based on a previous study by den Hertog *et al*.^21^. However, this threshold was established after analysis of only a limited number of patients and larger studies are needed to explore the use of alternative thresholds or alternative strategies (e.g. percent decrease). Sensitivity analysis of different thresholds could also be explored by combining datasets of individual similar studies.

In conclusion, this study confirms an association between a decrease in IP-10 levels during the first week of treatment in HIV positive patients and a bacteriological confirmation at diagnosis. Further work should explore the use of IP-10 kinetics in combination with other promising biomarkers to increase the sensitivity and identify the group of approximately 20% of patients who do not present with initially raised levels of circulating IP-10. This group of bacteriologically confirmed cases who do not present with significantly raised circulating IP-10 warrant more detailed investigation. Additionally, similar studies in settings with high levels of drug resistance should be a priority, as biomarker kinetics may be able to identify patients with drug resistance on inappropriate therapy much more rapidly than is possible with microscopy or culture. The measurement of IP-10 in other samples, such as urine, should also be explored^14^. In summary, the correlation of this responsive easily measured biomarker^13,21,34^ with culture conversion or the identification of specific patient populations warrants further study.
